# Anti‐Pseudomonas Aeruginosa Bacteriophage Loaded Electrospun Fibers for Antibacterial Wound Dressings

**DOI:** 10.1002/marc.202400744

**Published:** 2025-01-13

**Authors:** Tian Ju, Jixuan Li, Andrew Weston, Giovanni Satta, Sara Bolognini, Mariagrazia Di Luca, Simon Gaisford, Gareth R. Williams

**Affiliations:** ^1^ UCL School of Pharmacy University College London 29–39 Brunswick Square London WC1N 1AX UK; ^2^ Centre for Clinical Microbiology Royal Free Campus University College London Rowland Hill Street London NW3 2PF UK; ^3^ Department of Biology University of Pisa Via San Zeno 39 Pisa 56127 Italy

**Keywords:** bacteriophage, coaxial electrospinning, ethyl cellulose (EC), isothermal calorimetry, polyvinyl alcohol (PVA), polyvinylpyrrolidone (PVP), pseudomonas aeruginosa

## Abstract

Antimicrobial resistance poses a growing threat to public health globally. Multidrug resistant *Pseudomonas (P.) aeruginosa* is detected in many infected wounds and is very challenging to treat with antibiotics. An alternative to antibiotics is to use bacteriophages, highly specific viruses able to kill even resistant bacteria. This work incorporates anti‐*P. aeruginosa Neko* phages into monoaxial and coaxial electrospun fibers to explore their potential for treating infected wounds. Phages are blended with polyvinyl alcohol (PVA) solution and either processed directly into fibers or used as the core in coaxial electrospinning with polyvinylpyrrolidone (PVP) and PVP/ethyl cellulose (EC) shell solutions. Coaxial fibers stored at −20 °C show promising stability results, with negligible phage titer loss after 6 months of storage. Phage release can be controlled by varying the shell composition. Coaxial fibers with PVP as the shell (PVA/Su PVP + phage) demonstrate immediate release while fibers with PVP/EC as the shell (PVA/Su PVP/EC + phage) display extended‐release. The antibacterial efficacy of phage lysate and phage‐loaded fibers is studied by isothermal calorimetry and found to be unaffected by electrospinning. Thus, it appears that phage‐loaded electrospun fibers merit further investigation as potential wound dressing materials.

## Graphical abstract

Multidrug resistant *Pseudomonas (P.) aeruginosa* is detected in many infected wounds and is challenging to treat with antibiotics. As an alternative, this work incorporates anti‐*P. aeruginosa Neko* phages into electrospun fibres to explore their potential for treating infected wounds. Phage release could be controlled by varying the fibre composition. The antibacterial efficacy of the phages is unaffected by electrospinning.

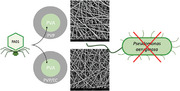

## 1 Introduction

Antibacterial resistance is a major global public health challenge, associated with an estimated 4.95 million deaths in 2019, disproportionately in low‐ and middle‐income countries.^[^ ^1^ ^]^ Since 2017, the World Health Organization (WHO) has released the Bacterial Priority Pathogens List (BPPL) to guide investment in research and form the basis for activities related to surveillance and control of antibacterial resistance. In the latest edition,^[^ ^2^ ^]^ *Pseudomonas aeruginosa* (in particular, if resistant to carbapenems) is classified among the *high‐group* pathogens, due to its significant burden in high‐income countries and certain regions such as central and eastern Europe and Central Asia.^[^ ^1^ ^]^

*P. aeruginosa* is one of the bacteria most commonly found in chronic and burn wounds.^[^ ^3^ ^]^ According to a study^[^ ^4^ ^]^ that analyzed data from 239 patients with wound infections, 57.9% of the species isolated were Gram‐negative bacteria, of which *P. aeruginosa* comprised 40.2%. Importantly, 12.9% of *P. aeruginosa* isolated showed resistance to no fewer than 6 antimicrobials. In another study^[^ ^5^ ^]^ that included 669 isolates from wounds, Gram‐negative bacteria were detected in 99.4% of samples and 81.5% of those were *Pseudomonas* spp. Among the isolated *Pseudomonas* spp strains, 82% were multidrug resistant. The emergence of antibiotic resistance is inevitable and increasingly concerning.

Bacteriophages (phages) are a potential antibacterial agent that could be used for treating antibiotic‐resistant infections.^[^ ^6^ ^]^ Phages are viruses that infect specific bacterial hosts.^[^ ^7^ ^]^ They replicate by penetrating the bacterial cell wall, forcing the host to produce viral components, and eventually the viral progeny burst out of the cell in a process called lysis. Phages are extremely species/strain‐specific, which means they only infect targeted bacterial species or even specific strains within a species.^[^ ^7^ ^]^ They have minimal impact on non‐target bacteria and other microorganisms, unlike broad‐spectrum antibiotics, which decrease the potential for inducing resistance. In addition, phage numbers scale according to the population of bacteria. When the host bacteria disappear, any residual phages will be eliminated by the human immune system without any negative impact on healthy tissue.^[^ ^8^ ^]^ In a Polish study,^[^ ^9^ ^]^ phages demonstrated therapeutic effects in patients with suppurative infections caused by multidrug‐resistant bacteria; 85.9% of patients showed full recovery and 10.9% were observed to enjoy transient improvement. However, a recent clinical trial (PhagoBurn) conducted in France and Belgium reported some negative results.^[^ ^10^ ^]^

PhagoBurn is one of the first phage therapies undergoing clinical trials. It comprises a polymeric wound dressing soaked in a phage cocktail containing twelve anti *P. aeruginosa* phages and was explored for treating *P. aeruginosa*‐infected burn wounds.^[^ ^10^ ^]^ The efficacy of the phage‐loaded wound dressing was compared with the standard of care (a 1% sulfadizaone silver emulsion cream). The phage‐loaded wound dressing was less effective than the cream but did result in a reduced bacterial burden in the infected wounds and provided a better safety profile, with fewer adverse events observed. However, the study report revealed that enhancing the stability of the phages through formulation improvement was required. Thus, incorporating phages into appropriate carriers to enhance their viability and stability, and also control their release profiles, is an issue of current concern. Electrospinning is one of the potential technologies that could be used to fulfill this unmet need.

Electrospinning is a technique that utilizes an electric force to solidify a polymer solution. It is easy to set up and cost‐effective. Monoaxial electrospinning comprises the simplest experiment, in which a polymer solution is dispensed through a single‐bore needle.^[^ ^11^ ^]^ Similarly, in coaxial electrospinning two polymer solutions are fed separately into a coaxial needle that is comprised of two capillary channels arranged in a concentric manner.^[^ ^11^ ^]^ Coaxial electrospinning enables controlled release of drugs and can prevent an initial burst of release. In addition, fragile bioactives, such as growth factors, DNA, and living organisms, can be encapsulated into the core of core‐shell nanofibers to avoid their exposure to harsh environments during the electrospinning process and storage.^[^ ^12^ ^]^

Phages have been successfully encapsulated into electrospun nanofibers on a number of occasions. Polyvinyl alcohol (PVA),^[^ ^13^, ^14^, ^15^ ^]^ polylactic‐co‐glycolic acid,^[^ ^16^ ^]^ polyethylene oxide (PEO),^[^ ^17^, ^18^ ^]^ polyvinylpyrrolidone (PVP),^[^ ^19^ ^]^ cellulose acetate,^[^ ^20^ ^]^ polycaprolactone,^[^ ^21^, ^22^, ^23^ ^]^ polyethlyene glycol,^[^ ^24^ ^]^ and polyester have all been utilized to encapsulate phages via monaxial, coaxial, and emulsion electrospinning, and also via surface immobilization. Phage‐loaded electrospun nanofibers have been developed for a range of different applications, such as food packaging, wound dressings, biosensors, and cell adhesive matrices. The antibacterial efficacy of phage‐loaded electrospun nanofibers against *P. aeruginosa*, *Staphylococcus aureus*,^[^ ^22^, ^23^, ^25^ ^]^ *Escherichia coli*, and *Mycobacterium tuberculosis* has been demonstrated in several studies. However, the rapid dehydration and high voltage applied during the monoaxial electrospinning process have been seen to cause great loss of phage viability (to as low as 1%–6%).^[^ ^13^ ^]^

This work encapsulated anti‐ *P. aeruginosa Neko* phages into monoaxial and coaxial fibers and explored their viability after being subjected to different electrospinning processes. PVA was chosen to fabricate the monoaxial fibers and the core of coaxial fibers, because PVA electrospinning can be performed using water as the solvent. It was hypothesized that water should reduce the loss of phage viability compared with polar organic solvents. The literature supports this idea: one study found that M13 phages had no structure change after exposure to apolar hexane for 8 h, while their structure showed a 10 fold contraction into rod‐like I‐forms after exposure to polar organic solvents.^[^ ^26^ ^]^ Another study tested phage viability after exposure to ethanol, isopropyl alcohol, tetrahydrofuran, and dimethyl sulfoxide.^[^ ^27^ ^]^ organic solvent for 20 min but showed morphology change and loss of titre (0.32–7.4 log_10_) when the concentration increased to 75%.

The effect of excipients (sucrose and alginate) on the viability of phages in the fibers was also investigated. For the core‐shell fibers, PVP or PVP/ethyl cellulose (EC) blends were selected to form the shell. PVP is very hydrophilic and is widely used for immediate‐release formulations.^[^ ^28^, ^29^, ^30^ ^]^ In contrast, EC is hydrophobic and can be blended with hydrophilic polymers (e.g. PVP^[^ ^31^, ^32^ ^]^ or PEO^[^ ^33^ ^]^) to control the drug release profile. The antibacterial efficacy of the phage‐loaded fibers was examined with isothermal calorimetry, which measures the heat production caused by the biological processes of a living organism. Its high sensitivity and ability to provide real‐time monitoring allow comparison of the growth curves of bacteria in the presence of phage lysate and phage‐loaded fibers, and thus show how the formulations influence antibacterial efficacy.

## 2 Methodology

### 2.1 Materials

PVA (molecular weight of 89000 – 98000, 99+% hydrolyzed, CAS No. 9002‐89‐5), PVP (K60, average Mw = 360000, CAS No. 9003‐39‐8), EC (viscosity 4 cP, 5% in toluene/ethanol 80:20(lit.), 48% ethoxyl, CAS No. 9004‐57‐3), alginic acid sodium salt (alginate, CAS No. 9005‐38‐3), phosphate‐buffered saline (PBS) tablets, sodium hydroxide (CAS No. 1310‐73‐2) and ethanol (CAS: 64‐17‐5) were all sourced from Sigma–Aldrich UK. Muller Hinton agar and tryptic soy broth (TSB) were purchased from Merck Millipore Ltd. Sucrose (CAS No. 57‐50‐1) and Triton X‐100 (TX100, CAS No. 9002‐93‐1) were obtained from Fisher Scientific and Union Carbide Company respectively. Storage Media (SM) buffer (pH 7) contains 100 mM sodium chloride (CAS No. 7647‐14‐5), 8 mM magnesium sulfate heptahydrate (CAS No. 10034‐99‐8), and 50 mM tris‐hydrochloride, which were acquired from Sigma–Aldrich UK, Fisher Scientific, and EMD Millipore Corp respectively. The pH value of the SM buffer was adjusted to 7 using sodium hydroxide. DNase I and proteinase K were purchased from ThermoFisher Scientific. A Zymo Research DNA Clean & Concentrator kit was obtained from Zymo Research. *Pseudomonas aeruginosa* (NCTC 12903) was purchased from TCS Biosciences. *P. aeruginosa Neko* phage was isolated from puddle water in La Spezia, Italy.

### 2.2 Electrospinning

#### 2.2.1 Monoaxial Fibers

For preparing placebo fibers without phages, 16.5% w/v PVA was dissolved in deionized water (DI water) with stirring at 85 °C for 20 min. Sucrose and alginate were chosen as excipients to improve the viability of phages in the fibers. The excipients and TX100 were blended with the PVA solution after it had cooled down. For phage‐loaded fibers, 16.5% w/v PVA was dissolved in DI water/phage lysate mixed at a v/v ratio of 80:20. PVA was first dissolved in DI water at 85 °C for 30 min. After the solution cooled down, the required excipients and TX100 were added. Finally, phage lysate (phage titer: 1–5 × 10^10^ plaque‐forming units (PFU)/mL) was added. The details are given in **Table** **1**. This method was adapted from our previous work.^[^ ^34^ ^]^

**Table 1:** Preparation of electrospinning solutions and the processing parameters employed to generate fibers.

Formulations	Preparation Methods	Flow Rate [mL h^−1^]	Distance [cm]	Voltage [kV]
Placebo Monoaxial fibers [core]				
PVA	330 mg of PVA was dissolved in 2 mL of DI water at 85 °C for 20 min. 3 µL of TX100 (0.15% v/v) was added after the solution cooled down.	0.3	14	12‐14
PVA/Su	0.1 m (68.5 mg) or 0.5 M (342.5 mg) sucrose was added to 2 mL of 16.5% w/v PVA solution and mixed for 30 min. 3 µL of TX100 (0.15% v/v) was added to the PVA solution after the solution cooled down.	0.3	14	12‐14
PVA/Alginate	Sodium alginate was dissolved in DI water overnight. 1.6 mL of 16.5% w/v PVA was blended with 0.4 mL of 4% w/v of sodium alginate solution for 30 min. 2 µL of TX100 (0.1% v/v) was added to the PVA/Alginate solution.	0.3	14	15‐17
PVA + phage	330 mg of PVA was dissolved in 1.6 mL of DI water at 85 °C for 20 min. 3 µL of TX100 (0.15% v/v) and 0.4 mL of phage lysate were added after the solution had cooled down.	0.3	14	14‐16
PVA/Su + phage	0.1 m (68.5 mg) or 0.5 m (342.5 mg) of sucrose and 330 mg of PVA were dissolved in 1.6 mL of DI water at 85 °C. 3 µL of TX100 (0.15% v/v) and 0.4 mL of phage lysate were added after the solution had cooled down.	0.3	14	13‐15
PVA/Alginate + phage	330 mg of PVA were dissolved in 1.6 mL of DI water at 85 °C. After the PVA solution had cooled down, 0.4 mL of 5% w/v of sodium alginate solution was mixed with it for 30 min. Later, 2.5 µL of TX100 (0.1% v/v) and 0.5 mL of phage lysate were added to the PVA/Alginate solution.	0.3	14	15‐17
Coaxial fibers (shell)				
PVP	2 g of PVP was dissolved in 20 mL of ethanol/water (16 mL/4 mL) overnight.	1.2	14	11‐14
PVP/EC	3 g of EC was dissolved in 20 mL of ethanol/water (16 mL/4 mL) overnight. 4 mL of 10% w/v PVP solution was blended with 1 mL of 15% w/v EC solution for 30 min before electrospinning.	1.2	14	11‐14
Name of coaxial formulations	Core	Shell		
PVA PVP	PVA	PVP		
PVA/Alginate PVP	PVA + Alginate	PVP		
PVA/0.1 m Su PVP	PVA + 0.1 M sucrose	PVP		
PVA/0.5 m Su PVP	PVA + 0.5 M sucrose	PVP		
PVA/0.1 m Su PVP/EC	PVA + 0.1 M sucrose	PVP + EC		

#### 2.2.2 Core–Shell Fibers

The core solution for coaxial spinning was the same as used to prepare monoaxial fibers. The shell consisted of PVP or PVP/EC. For the PVP shell, 10% w/v PVP was dissolved in ethanol/water (80:20 v/v). For PVP/EC, 15% w/v EC was first dissolved in ethanol/water (80:20 v/v); the EC solution was then blended with a 10% PVP solution in 80:20 v/v water/ethanol.

#### 2.2.3 Electrospinning Parameters

For monoaxial fibers, the polymer solution was loaded into a 1 mL syringe fitted with a metal spinneret (internal diameter (ID): 0.9 mm). The syringes were fixed on a syringe pump (78‐9100C, Cole–Parmer) which fed polymer solution at 0.3 mL h^−1^. An electric field was generated by a high voltage power DC supply (HCP35‐35000, FuG Elektronik) with the positive electrode connected to the spinneret and the grounded electrode connected to a metal plate covered with baking paper. The same electrospinning setup was used for the coaxial fibers, except two syringes and two syringe pumps were utilized. The core solution was loaded into a 1 mL syringe and the shell solution was loaded into a 5 mL syringe. The two syringes were connected to a metal coaxial spinneret (internal ID: 0.4 mm, outer ID:1.0 mm). The flow rate ratio of core (0.3 mL h^−1^) to shell (1.2 mL h^−1^) was set at 1:4. The voltage applied for the monoaxial fibers was 12–17 kV, and for coaxial fibers 11–14 kV. The temperature fell within the range of 22 ± 3 °C, and the relatively humidity was between 30 and 50%.

### 2.3 Bacterial Culture and Phage Propagation

*P. aeruginosa* (NCTC 12903) was subcultured in TSB overnight three times, at 37 °C. The bacteria culture underwent centrifugation (Sigma 3–16KL) at 10 000 rpm for 10 min, was washed with PBS three times, and then resuspended in 20% v/v glycerol/water. The bacteria were stored in cryovials at −80 °C. For the preparation of fresh bacterial culture, the frozen bacteria were thawed and grown in TSB at 37 °C.

Double‐layered agar assays were performed to harvest phages. 100 µL of bacteria culture (OD 0.4–0.7) was mixed with 50 µL of phage lysate and incubated for 10 min in a 7 mL plastic vial. Approximately 5 mL of soft Muller Hinton agar was then blended with the bacteria/phage mixture and vortexed for 10 s. The soft agar was poured on a solid agar plate and the plate was placed upside down in a 37 °C incubator overnight. The next day, 4 mL of SM buffer was pipetted on the agar plates and the plates were shaken for 30 min. The SM buffer along with soft agar was collected in centrifuge tubes and spun at 5000 rpm for 10 min. The supernatant (phage lysate) was removed and filtered through 0.22 µm syringe filters (Merck Millipore Ltd.). The filtered phage lysate was stored at 4 °C. The titer (1–5 × 10^10^ PFU mL^−1^) of the phage lysate was evaluated using a double‐layered agar assay. The plaques were counted manually.

### 2.4 One‐Step Growth Curve

Bacteria that entered the exponential growth phase (10^8^ CFU mL^−1^) were mixed with phages (10^7^ PFU mL^−1^) at a multiplicity of infection of 0.1 and then incubated at 37 °C for 10 min. This mixture underwent centrifugation (SciSpin Micro) at 10 000 rpm for 5 min to remove free phages. The supernatant was removed and the bacteria pellet was resuspended in 1 mL of TSB. The suspension was then transferred into 9 mL of TSB, and incubated at 37 °C under agitation (100 rpm). 20 µL of bacteria culture was removed at 15, 30, 45, 60, 70, 80, 90, 100, 110, 120, 150, and 180 min. Double‐layered spot assays were performed for phage enumeration. Approximately 5 mL of soft Muller Hinton agar was blended with bacteria (OD 0.4–0.7), vortexed for 10 s, and poured on a solid agar plate. 20 µL of bacteria culture underwent a serial dilution and 5 µL of diluted bacteria culture was dropped on the soft agar layer. After the drops had dried out, the plate was placed upside down in a 37 °C incubator overnight. The plaques were counted manually. This experiment was repeated in triplicate.

### 2.5 Phage genome Extraction and Analysis

The phage genome was extracted using procedures previously reported.^[^ ^35^ ^]^ Briefly, 20 µL of 10X DNaseI was added to 180 µL of phage lysate. Subsequently, 10 µL of DNaseI (1 U µL^−1^), RNA‐free, was added and incubated at 37 °C for 30 min. Then, 20 µL of 50 mM EDTA and 20 µL of 1% SDS were added. 10 µL of proteinase K (>600 U mL^−1^) was added, followed by an incubation at 55 °C for 45 min. For phage‐DNA purification, the Zymo Research DNA Clean & Concentrator kit was used according to manufacturer instructions. The genome was quantified using a NanoDrop Lite (ThermoFisher). The DNA samples were stored at −20 °C.

Illumina sequencing libraries were prepared using the Nextera Flex DNA library kit. The resulting raw sequences were uploaded to the BV‐BRC platform (https://www.bv‐brc.org/). Genome assembly was performed using the Unicycler tool (v0.4.8), which is available on the website, and the output was visually inspected to confirm the presence of a complete contig using the Bandage software (v 0.8.1).^[^ ^36^ ^]^ The resulting FASTA files were then processed through BLASTn (https://blast.ncbi.nlm.nih.gov/) to identify the closest matching phage genome in the NCBI database.

The genome of the best hit was downloaded. Genome alignments were performed using the EASYFIG (v2.1) tool to determine the sequence starting points, after which the phage genome FASTA files were manually corrected with the aid of UGENE (v49.1). The updated FASTA files were reuploaded on the BV‐BRC platform, where putative coding sequences (CDSs) were predicted using RASTtk (v1.3.0). This initial automated annotation was further refined manually by running BLASTp on each predicted protein. Functions were assigned to proteins based on homology to known proteins in the NCBI database.

In addition to the annotation gene by gene, the possible presence of antibiotic‐resistance or virulence genes was evaluated using ABRicate on the Galaxy platform (https://usegalaxy.org.au) using the ResFinder 4.1 database,^[^ ^37^ ^]^ VirulenceFinder 2.0,^[^ ^38^, ^39^ ^]^ CARD^[^ ^40^ ^]^ and the NCBI Antimicrobial Resistance Gene Finder 4.0.^[^ ^41^ ^]^

The genome information is deposited in GenBank, with accession number PQ628080.

### 2.6 Viability and Stability of Phages in the Electrospun Fibers

Approximately 2 mg of monoaxial fibers or 5 mg of coaxial fibers were immersed in 1 mL of SM buffer (pH 7) and placed in a 37 °C shaking incubator for 4 h. 50 µL of the sample was withdrawn and was serially diluted by factors of 10. The double‐layered agar assay was conducted to enumerate the phages released from 2 mg of fibers. The viability of phages in the fibers was expressed as the percentage ratio of viable phages to total phages. An example calculation is given in the Supporting Information. For evaluating the stability of phages in the electrospun fibers stored at room temperature (RT, 22 ± 3 °C), 4 °C, and −20 °C, the same method was applied. The samples were analyzed after being stored for 1 week, 1, 3, and 6 months. The phage titre (PFU) was plotted as a function of time. The experiments were repeated in triplicate and results are reported as mean ± standard deviation (SD).

### 2.7 Morphology

#### 2.7.1 Transmission Electron Microscopy (TEM)

Phage lysate was negatively stained with 2% phosphotungstic acid hydrate. The morphology was then observed with transmission electron microscopy (JOEL‐1400Flash instrument). The core–shell structure of the coaxial fibers was also explored with TEM (Philips CM120 Biotwin microscope). The TEM grids used were 3.05 mm copper grids with a formvar/carbon support film (300 mesh). The diameters of the phages (n = 10 objects) were measured using ImageJ.

#### 2.7.2 Scanning Electron Microscopy (SEM)

The morphology of the fibers was visualized with a bench‐top SEM (Phenom ProX, ThermoFisher Scientific). The samples were coated by gold sputtering and images were taken at an excitation voltage of 10 kV. The diameter of fibers (n = 100) was measured using ImageJ and the size distribution was plotted utilizing GraphPad Prism 10. The mean diameter (MD) and SD were calculated.

### 2.8 Thermal Analysis

The solvent loss and degradation temperature of the samples were examined with the aid of a Discovery thermogravimetric analyzer (TA Instruments). Approximately 3 mg of samples were placed in aluminum cups (TA instruments) and heated from 40 °C to 350–450 °C at a rate of 10 °C min^−1^, under a nitrogen flow rate of 25 mL min^−1^. Differential scanning calorimetry (DSC) thermograms were recorded with a Q2000 calorimeter (TA Instruments). Approximately 5 mg of sample was placed in a Tzero aluminum pan (TA Instruments) and heated from 40 °C to 200–300 °C at a rate of 10 °C min^−1^, under a nitrogen flow of 50 mL min^−1^. The results are shown in the Supporting Information.

### 2.9 X‐ray Diffraction (XRD)

The physical form was characterized with XRD (MiniFlex 600 diffractometer, RigaKu). A Cu Kα source (40 kV, 15 mA) generated X‐rays of 1.5418 Å wavelength. The samples were scanned over the 2θ angle range 5–50°, at a speed of 5° min^−1^. The results are presented in the Supporting Information.

### 2.10 Fourier Transform Infrared (FTIR) Spectroscopy

A Spectrum 100 instrument (PerkinElmer) was utilized to obtain FTIR spectra of the samples over the range from 4000 to 650 cm^−1^, with a resolution of 1 cm^−1^. An average of eight scans were taken for each spectrum. The results are illustrated in the Supporting Information.

### 2.11 Phage Release

Approximately 10 mg of monoaxial fibers or 25 mg of coaxial fibers were immersed in 5 mL of SM buffer (pH 7) and placed in a 37 °C shaking incubator. 50 µL aliquots were taken out at 1, 5, 10, 30, 60, 120, 240, and 360 min. The samples went through a ten‐fold serial dilution and then a double‐layered agar assay was performed to count the phages released from the fibers at different time points. The phage release curves comprise phage titer (PFU) versus time (min).

### 2.12 Antibacterial Activity

#### 2.12.1 Broth Dilution

The antibacterial efficacy of *Neko* phage lysate was tested using broth dilution. 200 µL of bacteria (10^7^ CFU mL^−1^), 200 µL of phage lysate (10^8^/10^7^/10^6^ PFU mL^−1^), 1 mL of TSB, and 0.6 mL of SM buffer were added in 7 mL plastic vials and vortexed for 15 s. 100 µL of the resultant mixed solution was transferred into 96 well plates. The plate was placed in a 37 °C incubator overnight. Optical density at *λ*_max_ = 600 nm was then measured on a UV spectrophotometer (Cary100, Agilent). The experiments were repeated in triplicate and the results are shown in the Supporting Information.

#### 2.12.2 Isothermal Calorimetry Assays

In order to investigate the antibacterial efficacy of *Neko* phages at different multiplicities of infection (MOI 100/10/1/0.1), the phage lysate was diluted to 10^9^, 10^8^, 10^7^, and 10^6^ PFU mL^−1^. After the OD 600 of the bacteria culture reached 0.4–0.5, it was diluted and the bacteria density set to be 10^7^ CFU mL^−1^. 50 µL of phage lysate, 50 µL of bacteria, 1.5 mL of tryptic soy agar, and 3.4 mL of SM buffer (pH 7) were added into 20 mL glass ampoules.

For testing the antibacterial efficacy of phage‐loaded fibers at MOI 100/10/1, ≈ 1, 10, or 100 mg of PVA/Su PVP + phage coaxial fibers were added into ampoules containing 50 µL of bacteria (10^7^ CFU mL^−1^), 1.5 mL of TSB, and 3.45 mL of SM buffer (pH 7). Similarly, for PVA/Su PVP/EC + phage coaxial fibers, 1.2, 12, and 120 mg of fibers were added to the ampoules; for the blend fibers, 1.1, 11, and 110 mg of fibers were added. The ampoules were sealed with metal lids, vortexed for 10 s, and incubated in a thermal activity monitor (TAM2277, TA Instruments) at 37 °C for 30 min before recording the heat flow. The heat flow (power, mW) was plotted against time (h). All the experiments were repeated in triplicate and one representative dataset is displayed.

### 2.13 Statistical Analysis

The data were processed statistically using GraphPad Prism 10. Any statistically significant differences between the two means were determined using a *t*‐test. *p* < 0.05 was considered significant.

## 3 Results and Discussion

### 3.1 Characterization and Genome Analysis of the *Neko* Phage

**Figure 1:**
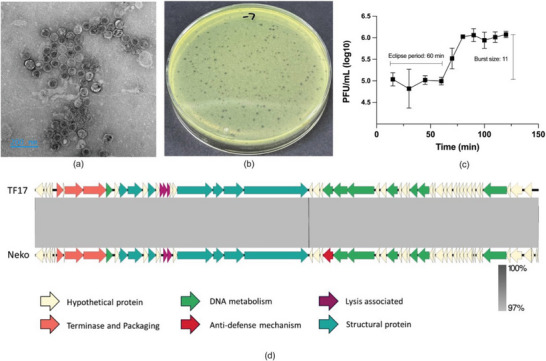
a) TEM image of *Neko* phages (scale bar 200 nm). b) Plaques of *Neko* on a double‐layered agar plate with *P. aeruginosa* (NCTC 12903). c) One‐step growth curve of *Neko* phages (n = 3). d) Genome organization of *Neko* phage compared with phage vB_PaeP_TF17 (NCBI: txid2530025). The arrows indicate annotated ORFs as follows: green arrows indicate ORFs associated with DNA metabolism‐associated proteins, orange ORFs encode for terminase and packaging proteins, and yellow ORFs for hypothetical proteins; purple ORFs denote lysis‐associated proteins; red are ORFs that encode for anti‐defence mechanism‐associated proteins; in turquoise are structural proteins. The color intensity of the bands between two compared sequences indicates BLASTn percentage similarity.

The morphology of *Neko* was visualized by TEM (**Figure** **1**a). The mean diameter of the capsid was 66 ± 3 nm. *Neko* can form small, clear, and round plaques on MHA double‐layered agar plates (Figure 1b), with the diameter of the plaque being ≤ 1 mm. The one‐step growth curve is presented in Figure 1c. The eclipse period of *Neko* phage is 60 min and the burst size is 11.

From its genome analysis (Figure 1d), the phage is a Podovirus, belonging to the *Caudoviricetes* family with a genome length of 43541 bp. *Neko* showed the highest similarity with *P. aeruginosa* phage *vB_PaeP_TF17* (NCBI: txid2530025), with which it shares 100% and 97.18% of coverage and identity, respectively. The phage is a strictly virulent virus and no genes encoding proteins associated with acquired bacterial antibiotic resistance, virulence factors, or toxins were predicted, suggesting that *Neko* is a safe candidate for phage therapy.

### 3.2 Phage Viability After Electrospinning

**Figure 2:**
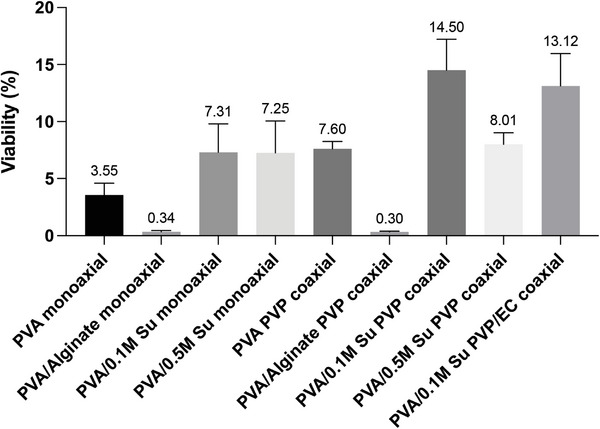
Phage viability after monoaxial and coaxial electrospinning with different excipients (n = 3).

Phage viability after electrospinning is defined as the percentage of phages that maintain infectivity against their hosts after being subjected to electrospinning. Maintaining viability can be a significant challenge. Salalha et al.^[^ ^13^ ^]^ separately encapsulated T4, T7, or λ phages in PVA monoaxial fibers and found viability after electrospinning to be 1%, 2%, and 6%, respectively. Korehei et al.^[^ ^17^ ^]^ utilized suspension, emulsion, and coaxial electrospinning to incorporate T4 phages in PVP fibers. The titre of the original phage stock was 10^8^ PFU mL^−1^. After electrospinning, the phage titer was at 10^3^ for suspension electrospinning and at 10^6^ for emulsion electrospinning, whereas the titre was 10^8^ with coaxial electrospinning. Phage viability is vital to ensure therapeutic efficacy, and thus decreasing the phage titre loss caused by electrospinning is essential. In order to improve phage viability after electrospinning and investigate the impact of the electrospinning process and excipients, phages were processed by monoaxial and coaxial spinning with different excipients: alginate and sucrose. The percentage viability obtained with the different formulations is shown in **Figure** **2**.

Even though alginate has been used extensively for formulating phages,^[^ ^42^, ^43^, ^44^ ^]^ the results demonstrated that alginate is not a suitable excipient here. Phage viability in fibers with alginate was much lower in those without alginate: phage viability in PVA monaxial fibers without any excipients was 3.55%, while in PVA/Alginate monaxial fibers it was 0.34%, and in PVA/Alginate‐PVP coaxial fibers 0.30%. Sucrose (Su) increased phage viability after electrospinning twofold (PVA monoaxial: 3.55%, PVA/0.1 m Su monoaxial: 7.31%). However, increasing the concentration of sucrose in the polymer solution did not enhance phage viability further after electrospinning (PVA/0.5 m Su monoaxial: 7.25%). Sucrose improves phage viability by stabilizing intraprotein hydrogen bonds and water replacement^[^ ^45^ ^]^ when phages undergo dehydration during electrospinning.

Coaxial electrospinning increased phage viability twofold in some formulations. Phage viability in the PVA/PVP coaxial system was 7.62% (cf. monoaxial PVA 3.55%). Similarly, phage viability in PVA/0.1 m Su PVP fibers was 14.50%, and in PVA/0.1 m Su monoaxial fibers 7.31%. However, coaxial electrospinning did not always enhance phage viability in the fibers. For example, phage viability in PVA/Alginate PVP coaxial fibers (0.30%) and PVA/0.5 m Su PVP coaxial fibers (8.01%) was comparable to the corresponding monoaxial fibers without a PVP shell (PVA/Alginate monoaxial: 0.34%, PVA/0.5 m Su monoaxial: 7.25%). The addition of EC to the shell did not have a significant impact on phage viability. Phage viability in PVA/0.1 M Su PVP coaxial fibers was 14.50% and in coaxial fibers with EC (PVA/0.1 m Su PVP/EC coaxial fibers) 13.12%.

Based on the above results, the formulations with alginate and 0.5 m sucrose were not taken forward, because they resulted either in low phage viability or no improvement of viability. The systems using a PVA solution with 0.1 m sucrose were found to be optimal in terms of phage viability and thus were explored in more detail.

### 3.3 Phage Stability in the Fibers

**Figure 3:**
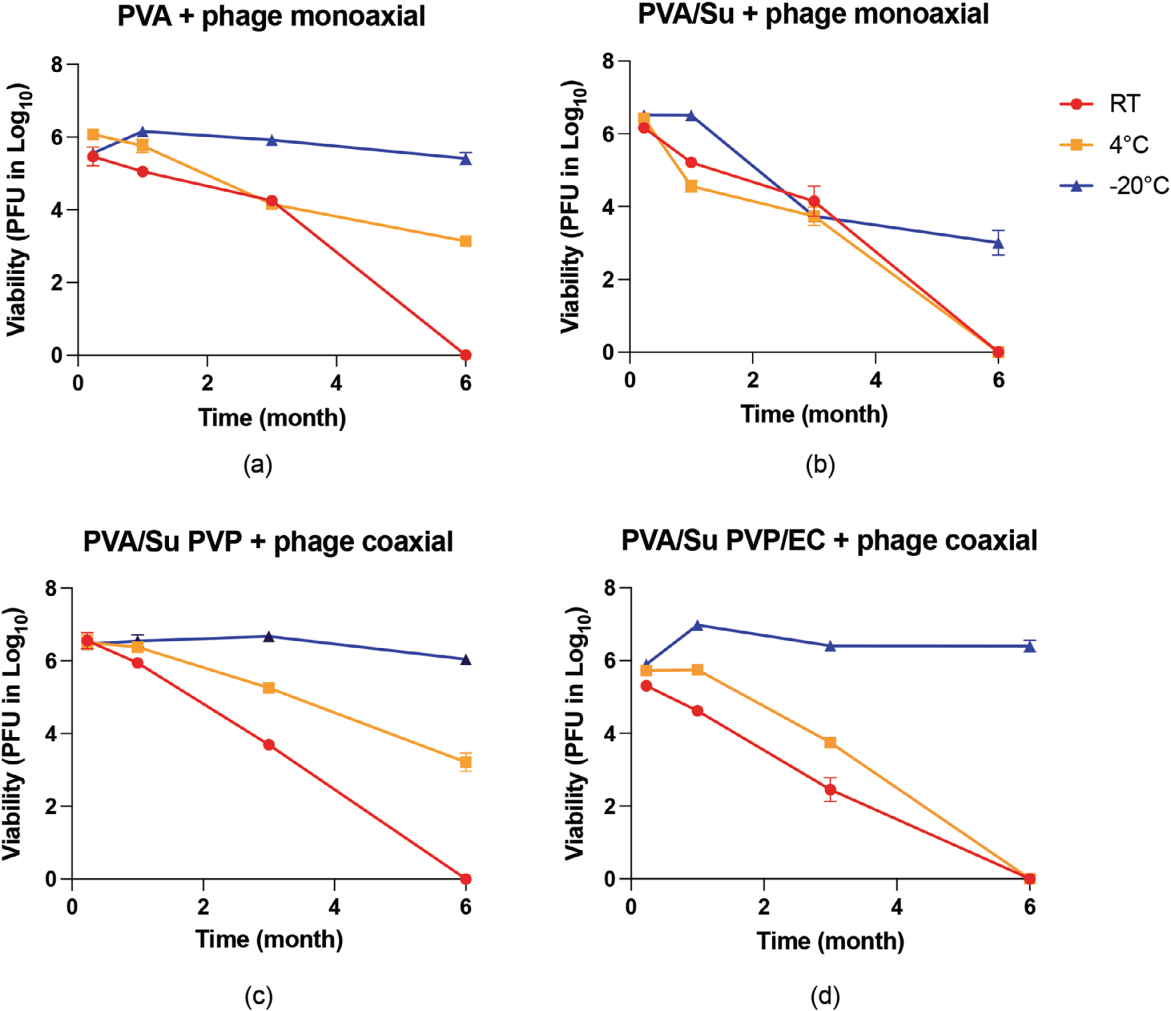
Phage viability in a) PVA + phage monoaxial, b) PVA/Su + phage monoaxial, c) PVA/Su PVP + phage coaxial, and d) PVA/Su PVP/EC + phage coaxial fibers stored at −20, 4 °C and room temperature (RT) over 6 months (n = 3).

Testing phage stability in the fibers is important for understanding how the phage titre varies with time and the selection of proper storage and transportation conditions. Here, phage stability in the monaxial and coaxial fibers after storage at −20, 4 °C, and RT over 6 months (**Figure** **3**) was examined. For PVA monoaxial fibers with and without sucrose (Figure 3a,b), it appeared that the presence of sucrose did not have a protective effect on the phages, and even reduced phage stability in the monoaxial fibers. The phage titre in PVA + phage monoaxial fibers (without sucrose) stored at −20 °C had no noticeable reduction after 3 months of storage and had less than 1 log reduction after 6 months. However, the phage titre in the PVA/Su + phage monoaxial fibers (with sucrose) stored at −20 °C showed more than 2 log reduction after 3 months of storage and more than 3 log reduction after 6 months. There was a notably greater loss in the phage titre when both monoaxial formulations were stored at 4 °C or RT.

For coaxial fibers (PVA/Su PVP + phage and PVA/Su PVP/EC + phage coaxial fibers, Figure 3c,d) stored at −20 °C, the phages maintained full viability for 3 months, while the titre had less than 1 log loss after 6 months of storage. For coaxial fibers stored at 4 °C and RT, the phage titer had either more than 3 log reduction or lost viability completely after 6 months. The coaxial fibers contained sucrose in the core to improve phage viability. Unlike PVA/Su + phage monoaxial fibers, sucrose did not contribute to any obvious titre loss when the coaxial fibers were stored at −20 °C. Moreover, the addition of EC in the shell did not have any obvious impact on stability. Phage stability in the coaxial fibers exhibited similar trends to the PVA + phage monoaxial fibers, which infers that coaxial fibers do not always result in improvements in long‐term stability.

Phage stability in electrospun fibers has been studied by others. Salalha et al.^[^ ^13^ ^]^ loaded T4, T7, and λ phages separately into a PVA solution and fabricated monoaxial fibers before testing the viability of phages in fibers stored at RT, 4, −20, and −55 °C for 3 months. Phages lost viability completely after one month of storage at RT; some loss of viability was also observed when the phage‐loaded fibers were stored at 4 °C for 3 months. However, the phages maintained full viability after being stored at −20 °C, and −55 °C. In another study, Korehei et al.^[^ ^17^ ^]^ fabricated coaxial electrospun fibers using phage/buffer suspension as the core and PEO solution as the shell. The phage‐loaded fibers stored at 20 °C had more than 8 log reduction of phage titre, whereas those stored at 4 °C and ‐20 °C exhibited negligible titre reduction over 4 weeks of storage. This work is thus consistent with previous reports and shows that ‐20 °C is a suitable temperature for long‐term storage of phage‐loaded electrospun fibers.

### 3.4 Morphology of Electrospun Fibers

**Figure 4:**
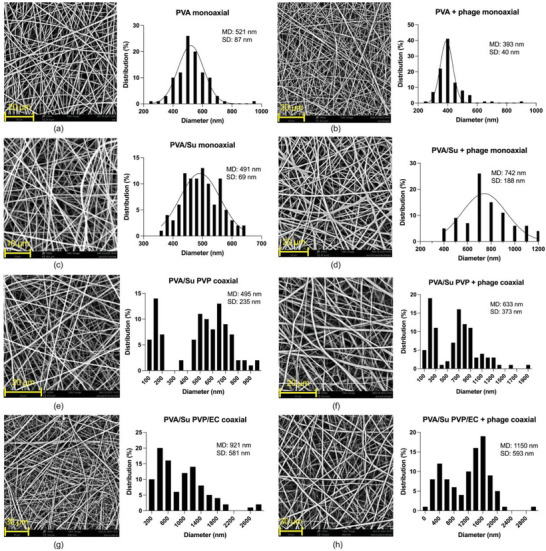
SEM images and size distribution of a) PVA monoaxial, b) PVA + phage monoaxial, c) PVA/Su monoaxial, d) PVA/Su + phage monoaxial, e) PVA/Su PVP coaxial, f) PVA/Su PVP + phage coaxial, g) PVA/Su PVP/EC coaxial, h) PVA/Su PVP/EC + phage coaxial electrospun fibers (n = 100).

**Figure 5:**
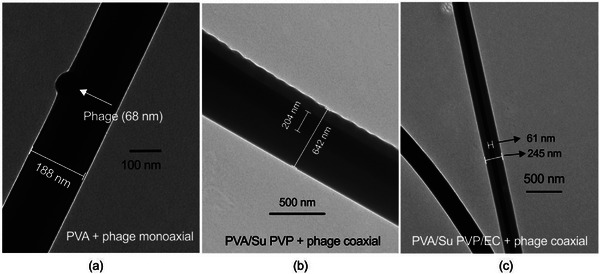
TEM images of a) a phage encapsulated in PVA monoaxial fibers, and the core–shell structure of b) PVA/Su PVP + phage and c) PVA/Su PVP/EC phage coaxial fibers.

The morphology and size distribution of phage‐loaded fibers and placebo fibers without phages are presented in **Figure** **4**. SEM images demonstrate that all the fibers are cylindrical and have a smooth surface. The monoaxial fibers are normally distributed in size, and have a diameter ranging between 393 and 742 nm. The phages were successfully encapsulated in monoaxial fibers, as can be seen by TEM (**Figure** **5**a). PVA + phage monoaxial fibers (393 nm) have a smaller diameter than pure PVA fibers (521 nm). The decreased diameter of phage‐loaded PVA monoaxial fibers resulted from increased conductivity induced by phage lysate (which contains phages, NaCl, MgSO_4_•7H_2_O, Tris‐HCl, and DI water). A polymer solution with higher charge density can form a jet that is easier to stretch when a high voltage is applied, thus promoting the formation of thinner fibers.^[^ ^46^ ^]^ Sucrose did not have a significant effect on the diameter of the fibers. PVA/Su monoaxial fibers (491 nm) had a similar diameter to PVA monoaxial fibers (521 nm). Differing from PVA + phage monoaxial fibers, adding phage lysate to PVA/Su solution (PVA/Su + phage monoaxial: 742 nm) led to an increased diameter. The increased diameter possibly results from dispensing more mass of solute per unit time. In a previous study, Salalha et al.^[^ ^13^ ^]^ prepared phage‐loaded PVA nanofibers with a diameter of 160 nm. Sarhan and Azzazy^[^ ^14^ ^]^ fabricated monoaxial fibers using PVA solution, honey, chitosan, bee venom, and phages and obtained mean diameters of ≈600 nm. The results reported here are hence in line with the literature.

Compared with the monoaxial fibers, the coaxial fibers generally had larger diameters. The core–shell structure of phage‐loaded coaxial fibers can be seen in the TEM images given in Figure 5b,c. All the coaxial fibers (Figure 4e–h) had a bimodal size distribution. Bimodal distributions have previously been reported when fabricating polyacrylonitrile,^[^ ^47^ ^]^ graphene oxide/nylon‐6 composite,^[^ ^48^ ^]^ and poly(ε‐caprolactone)^[^ ^49^, ^50^ ^]^ fibers, for instance. The formation of a bimodal distribution may arise due to splaying of the jet during unstable jet elongation and loss of solvent.^[^ ^11^ ^]^ It is typically observed in solutions that are highly concentrated, as well as under high field strength.

Though using one mean for a set of bimodally distributed data cannot reflect the real value, single values are reported here to simplify the comparison between different formulations. Phage‐loaded coaxial fibers had a larger diameter than placebo fibers. For instance, the mean diameter of PVA/Su PVP fibers was 495 nm, while that of PVA/Su PVP + phage fibers was 633 nm. Furthermore, adding EC in the PVP shell contributed to a markedly increased diameter. PVA/Su PVP/EC fibers (921 nm) were almost twice as thick as the PVA/Su PVP fibers (495 nm). Likewise, the diameter was 1150 nm for PVA/Su PVP/EC + phage fibers and 633 nm for PVA/Su PVP + phage fibers. These values are comparable to a previous study^[^ ^18^ ^]^ in which the T4 phage was entrapped in PEO or PEO/cellulose diacetate coaxial fibers (which had diameters from 1.35 to 2.48 µm).

### 3.5 Physical Form Characterisation

The placebo fibers underwent a detailed physical form characterization using TGA, DSC, XRD, and IR spectroscopy. TGA thermograms (Figure S1, Supporting Information) and DSC data (Figure S2, Supporting Information) indicated the presence of some residual water in the formulations (mass loss below 100 °C in TGA, with a corresponding solvent loss endotherm in DSC). The fibers appeared amorphous by XRD (Figure S3, Supporting Information), while some crystalline features of PVA and sucrose are seen in DSC, presumably as a result of recrystallisation upon heating. IR spectra (Figure S4, Supporting Information) confirm the presence of the various components of the fibers.

### 3.6 In Vitro Phage Release

**Figure 6:**
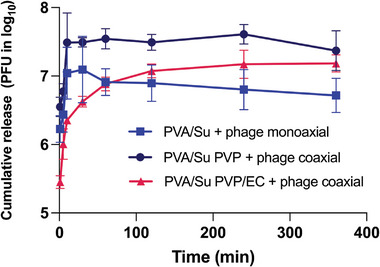
The in vitro phage release profiles of PVA/Su + phage monoaxial fibers (light blue), PVA/Su PVP +phage coaxial fibers (dark blue), and PVA/Su PVP/EC + phage coaxial fibers (pink). The experiments were conducted in triplicate.

The phage release profiles are presented in **Figure** **6**. PVA/Su + phage monoaxial fibers (light blue) and PVA/Su PVP + phage coaxial fibers (dark blue) exhibited an initial burst release: most phages were released within 10 min. The release curves then started to plateau. The burst release of the PVA/Su + phage monoaxial fibers is attributed to the hydrophilicity of PVA, the high‐surface‐area‐to‐volume ratio, and the high porosity of electrospun fiber mats. The PVA/Su PVP + phage coaxial fibers contain a PVA/Su + phage core, with the addition of a PVP shell. This gives a similar phage release profile to the monoaxial fibers, suggesting that the PVP shell does not have an impact on the release. Because PVP is very hydrophilic and can dissolve rapidly into water, the PVP shell dissolves in the SM buffer quickly and leaves only the PVA/Su + phages core (PVA does not dissolve in water at RT). Thus, the phage release profile is mainly determined by the core of the fibers.

To prevent the initial burst release of phages from the electrospun fibers, EC was mixed with PVP in the shell. This resulted in the phages being gradually released over a period of 2 h (Figure 6, pink curve). Due to the hydrophobicity of ethyl cellulose, the PVP/EC shell did not fully dissolve in the SM buffer, thus creating a barrier that slowed down buffer ingress into the core of fibers and hindered phages from diffusing out of the fibers. Moreover, because the PVA/Su PVP/EC + phage fibers (1150 nm) are thicker than PVA/Su PVP + phage (633 nm), more time was required for the phages to diffuse out of the fibers.

Only a few studies have reported the phage release profiles of electrospun fibers. Korehei and Kadla^[^ ^18^ ^]^ encapsulated T4 phage/buffer suspension (core) in PEO and PEO/CDA (shell) fibers using coaxial electrospinning. For the PEO/T4 phage fibers, almost 100% of the phage content was released within 30 min owing to the hydrophilicity of PEO. To reduce the release rate, hydrophobic CDA was blended with PEO. This slowed release but most phages were still released within 1 h. Blending hydrophilic and hydrophobic polymers for prolonging the release of phages proved to be successful both in this work and Korehei's, with the systems reported here offering more extended release than the literature reports.

### 3.7 Isothermal Calorimetry Assays

In isothermal calorimetry, the heat released or absorbed during biological processes is measured directly. The technique has a long history of use in microbiology but has surprisingly only been used to a limited extent to study the interaction between bacteria and phages. The high sensitivity of the technique means that bacterial activity in the presence of phage lysate and phage‐loaded electrospun fibers can be measured in real time, showing the rate at which the phages arrest bacterial growth. The antibacterial efficacy of phage lysate was explored first to serve as a reference. By comparing the bacteria growth curves in the presence of phage lysate with the growth curves in the presence of phage‐loaded electrospun fibers, the impact of the formulations on phage activity could be quantified. The growth parameters, including the height of the first (*H*_1st_), second (*H*_2nd_), and maximum (*P*_max_) peaks and their corresponding time of appearance (*t*_1st_, *t*_2nd_, and *t*_max_) are summarized in **Table** **2**.

**Table 2:** Growth parameters of *P. aeruginosa* incubated with phage lysate and phage loaded fibers.

MOI	0 [PC/Placebo]	0.1	1	10	100
Phage solution – Figure 7a					
*H*_1st_ (h)	2.6 ± 0.3	2.5 ± 0.3	2.1 ± 0.2	0.9 ± 0.1	1.6 ± 0.1
*t*_1st_ (mW)	6.4 ± 0.6	6.9 ± 0.5	7.1 ± 0.6	7.1 ± 1.0	13.9 ± 0.3
*H*_2nd_ (h)	/	/	/	1.3 ± 0.1	/
*t*_2nd_ (mW)	/	/	/	11.8 ± 0.5	/
*P*_max_ (h)	2.6 ± 0.3	2.5 ± 0.3	2.1 ± 0.2	1.3 ± 0.1	1.6 ± 0.1
*t*_max_ (mW)	6.4 ± 0.6	6.9 ± 0.5	7.1 ± 0.6	11.8 ± 0.5	13.9 ± 0.3
PVA/Su PVP + phage fibers – Figure 7b					
*H*_1st_ (h)	2.5 ± 0.2	/	1.9 ± 0.3	1.0 ± 0.3	1.6±0.2
*t*_1st_ (mW)	7.5 ± 1.0	/	7.2 ± 0.4	7.8 ± 0.6	15.4±0.1
*H*_2nd_ (h)	/	/	/	1.3 ± 0.2	/
*t*_2nd_ (mW)	/	/	/	13.5 ± 1.6	/
*P*_max_ (h)	2.5 ± 0.2	/	1.9 ± 0.3	1.3 ± 0.2	1.6 ± 0.2
*t*_max_ (mW)	7.5 ± 1.0	/	7.2 ± 0.4	13.5 ± 1.6	15.4 ± 0.1
PVA/Su PVP/EC + phage fibers – Figure 7c					
*H*_1st_ (h)	2.5 ± 0.1	/	2.1 ± 0.2	1.7 ± 0.1	1.0 ± 0.1
*t*_1st_ (mW)	6.9 ± 0.1	/	7.6 ± 0.2	7.4 ± 0.4	8.4 ± 1.0
*H*_2nd_ (h)	/	/	/		1.7 ± 0.5
*t*_2nd_ (mW)	/	/	/		17.0 ± 1.2
*P*_max_ (h)	2.5 ± 0.1	/	2.1 ± 0.2	1.7 ± 0.1	1.7 ± 0.5
*t*_max_ (mW)	6.9 ± 0.1	/	7.6 ± 0.2	7.4±0.4	17.0 ± 1.2
Mixture fibers – Figure 7d					
*H*_1st_ (h)	2.4 ± 0	/	2.0 ± 0.2	1.3 ± 0.1	1.9 ± 0.2
*t*_1st_ (mW)	7.1 ± 0.2	/	7.6 ± 0.6	7.8 ± 0.7	16.3 ± 0.4
*H*_2nd_ (h)	/	/	/	1.2 ± 0.1	/
*t*_2nd_ (mW)	/	/	/	11.6 ± 0.9	/
*P*_max_ (h)	2.4 ± 0	/	2.0 ± 0.2	1.3 ± 0.1	1.9 ± 0.2
*t*_max_ (mW)	7.1 ± 0.2	/	7.6 ± 0.6	7.8 ± 0.7	16.3 ± 0.4

MOI: multiplicity of infection (the ratio of phages to bacteria). PC (MOI 0): positive control, which only contains bacteria and growth media. Placebo (MOI 0): contains placebo fibers without phages, bacteria, and growth media. *H*_1st_: height of the first peak, *t*_1st_: time that the first peak appears, *H*_2nd_: height of the second peak, *t*_2nd_: time that the second peak appears, *P*_max_: maximum peak, *t*_max_: the time when the maximum peak appears. The experiments were conducted in triplicate. The mean and standard deviation are presented in the table. The difference with *P* < 0.05 was considered significant.

**Figure 7:**
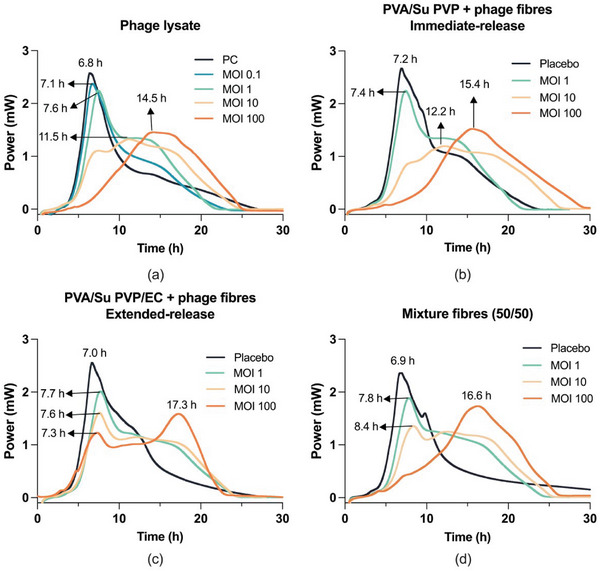
Calorimetric growth curves for *P. aeruginosa* in the presence of a) a *Neko* phage lysate, b) PVA/Su PVP + phage fibers (immediate‐release formulation), c) PVA/Su PVP/EC + phage fibers (extended‐release formulation), d) a mixture of fibers (50% PVA/Su PVP + phage fibers and 50% PVA/Su PVP/EC + phage fibers). Different ratios of phages to bacteria (MOI 0.1/1/10/100) were tested. Positive control (PC) only contains bacteria and growth media. Placebo contains bacteria, a growth media, and placebo fibers without phages. The experiments are repeated in triplicate. One representative set of data is shown.

The metabolic activity of bacteria releases heat, which consequently results in exothermic growth curves. The gradients and peak maxima of the curves can be used as indicators of the kinetics of growth. For *P. aeruginosa* incubated with phage lysate (**Figure** **7**a), the positive control without phage added (*P*_max_: 2.6 mW, *t*_max_: 6.4 h) and with phages at MOI 0.1 (*P*_max_: 2.5 mW, *t*_max_: 6.9 h) had only one main peak and the shape of the growth curves was similar (*P* > 0.05). For MOI 1 (*P*_max_: 2.1 mW, *t*_max_: 7.1 h), the shape of the growth curve started to change: a sharp peak followed by a broad hump can be seen. The shape of the growth curve at MOI 10 changed greatly, with a peak at an intermediate time accompanied by two shoulder peaks. The first peak appeared at 7.1 h and the corresponding power output was 0.9 mW. The *P*_max_ for MOI 10 (1.3 mW) was much smaller than MOI 1 (2.1 mW) (*P* < 0.05) and the *t*_max_ was delayed (MOI 10: 11.8 h, MOI 1: 7.1 h, *P* < 0.05). The shape of the growth curve for MOI 100 differed from other curves and comprised only one peak. In comparison with MOI 10, MOI 100 had a longer *t*_max_ (13.9 h, *P* < 0.05), but its *P*_max_ (1.6 mW) was slightly higher than MOI 10 (1.3 mW*, P* < 0.05). Adding phage lysate to the bacteria culture did not notably delay the onset time of heat production, which infers that phages are not able to delay the growth of the bacteria. However, as the MOI increased to 10 and 100, *t*_max_ extended and *P*_max_ decreased significantly. This indicates that the phages start to disrupt bacterial growth at these concentrations. The antibacterial efficacy of phage lysate was also tested using the broth dilution method. The results are shown in Figure S5 (Supporting Information). The trends in the data here are much less clear than in the calorimetry experiment, confirming the latter to be more appropriate for exploring the properties of the fibers.

PVA/Su PVP + phage fibers (immediate‐release) and PVA/Su PVP/EC + phage fibers (extended‐release) illustrated different phage release profiles and thus were selected for investigating how this affected the growth of bacteria. Figure 7b shows the growth curves of bacteria in the presence of an immediate‐release formulation (PVA/Su PVP + phage fibers). The mass of fibers was calculated based on the phage viability in the fibers. 1, 10, or 100 mg of fibers was added to give final MOIs of 1, 10, and 100. The values of *P*_max_ and *t*_max_ for placebo fibers (*P*_max_: 2.5 mW, *t*_max_: 7.5 h) were comparable to the positive control (*P*_max_: 2.6 mW, *t*_max_: 6.4 h) (*P* > 0.05), which signifies that the placebo formulation did not affect bacterial growth. The shape of the growth curves and values of *P*_max_ and *t*_max_ for PVA/Su PVP + phage fibers were close to the phage lysate at the same MOI (*P* > 0.05, except *t*_max_ for MOI 100), demonstrating that the formulation process did not have a noticeable influence on antibacterial efficacy. With the lysate, phages were in contact with bacteria immediately after addition to the ampoule. Similarly, for PVA/Su PVP + phage fibers, most phages were released into the surrounding medium within 10 min. As for the lysate, phages released from the formulation could quickly infect bacteria, and hence growth curves for phage lysate and PVA/Su PVP + phage fibers were similar.

The bacteria growth curves in the presence of the extended‐release formulation (PVA/Su PVP/EC + phage fibers) are given in Figure 7c. As for the immediate‐release formulation, the placebo extended‐release formulation (*P*_max_: 2.5 mW, *t*_max_: 6.9 h) did not have any influence on bacterial growth (*P* < 0.05). The shape of the growth curves, *P*_max_, and *t*_max_ at MOI 1 were close to phage lysate and the immediate‐release formulation at the same MOI. However, they differed from phage lysate and the immediate‐release formulation from MOI 10. The growth curve at MOI 10 for the extended‐release formulation had one sharp peak followed by a broad hump, which was similar to the shape of MOI 1 but with a lower *P*_max_. The growth curve at MOI 100 formed a concave shape with two peaks on both sides. The *t*_max_ (17 h) of the extended‐release formulation was slightly later than phage lysate at MOI 100 (13.9 h*, p* < 0.05) and the immediate‐release formulation (15.4 h, *p* < 0.05).

At MOI 1, there is only a small number of phages (5 × 10^5^ PFU) present and it does not take long to release them into the surrounding medium. In addition, the growth curves for phage lysate at MOI 0.1 and MOI 1 did not differ much (*p* > 0.05). Thus, even though the EC‐containing formulation gradually releases the phages, no obvious changes in the growth curve at MOI 1 between the extended‐release formulation and phage lysate were seen. However, when the MOI was higher (MOI 10: 5 × 10^6^ PFU and MOI 100: 5 × 10^7^ PFU), the shape of the growth curves for phage lysate changed markedly. For the EC extended‐release formulations, the phages were stored in the fibers and gradually released in the surrounding medium. Hence, the concentration of phages in the medium is relatively low in the first few hours. As a result, the phages are not able to reduce the growth rate of bacteria, resulting in the peaks at ≈7.5 hours at MOI 10 and 100 and consequently leading to different shapes of the growth curves compared to phage lysate and the immediate‐release formulation. In order to confirm the changes in growth curves were a consequence of EC extending the release time, a mixture (50/50 w/w) of the immediate‐release formulation and the extended‐release formulation was explored.

For the fiber mixture (Figure 7d), the growth curve at MOI 1 was similar to all the other formulations (*p* > 0.05). The growth curve at MOI 10 had a peak followed by a broad hump, like the growth curve for the extended‐release formulation but with a lower *P*_max_ value. The growth curve at MOI 100 had one main peak at 16.6 h with a small hump at 7 h. The growth curves of MOI 10 and 100 for the mixed fibers thus share the characteristic features of both formulations, suggesting that the growth of bacteria is affected by the phage release profiles of the formulations. This confirms that the changes in the growth curves are caused by EC extending the release of the phages.

It is clear that the phage lysate is not able to fully suppress the growth of bacteria over 24 h, but it can decrease the bacteria growth rate. Placebo formulations (containing placebo fibers without phages, bacteria, and a growth media) did not affect the growth of bacteria. The immediate‐release formulation (PVA/Su PVP + phage fibers) had similar growth curves as phage lysate. This indicates that these fibers successfully encapsulated the phages without affecting their antibacterial efficacy. The extended‐release formulation (PVA/Su PVP/EC + phage) presented similar growth curves as phage lysate and the immediate‐release formulation at MOI 1. However, when the total number of phages in the formulation was high (MOI 10 and 100), the extended‐release formulation could not reduce the bacteria growth rate in the first few hours due to the low initial phage concentration, consequently contributing to different growth curves.

Some previous studies explored the antibacterial efficacy of anti *E. coli*,^[^ ^51^, ^52^, ^53^ ^]^ *Proteus mirabilis*,^[^ ^53^ ^]^ and *S. aureus* ^[^ ^54^ ^]^ phages by isothermal microcalorimetry. These studies demonstrate that as the concentration of phages increases, *P*_max_ decreases. When the concentration of phages is high enough, the phage lysate can inhibit the growth of bacteria for a period of time. In this work, although the phage lysate was not able to fully supresses the growth of bacteria over 24 hours, the assays proved that the phage‐loaded immediate‐release formulation could successfully encapsulate phages without affecting their antibacterial efficacy. Finding more potent phages or utilizing phage‐antibiotic combinations for treating bacterial infections could be explored in the future to develop more effective formulations for clinical applications.

The formulations developed in this work successfully encapsulated anti *P. aeruginosa Neko* phages in electrospun fibers and retained their infectivity against *P. aeruginosa*. The excellent phage stability in the fibers enables long‐time storage. Electrospun fibers exhibit appealing properties for wound dressings: high porosity that allows gas exchange, good adaptability to the wound contour, and controlled delivery of antibacterial agents. The phage‐loaded electrospun fibers thus have the potential to be used as wound dressings for treating antibiotic‐resistant infections after the selection of appropriate active phages.

## 4 Conclusion

This study explored the possibility of using phage‐loaded electrospun fibers for treating wounds infected with *P. aeruginosa*. Anti *P. aeruginosa Neko* phages were incorporated in monoaxial and coaxial fibers. Coaxial fibers generally resulted in better phage viability than monoaxial fibers. The addition of sucrose (Su) improved the viability of phages, likely by stabilizing hydrogen bonds and replacing water lost during the electrospinning process, but alginate did not result in the same effects. Coaxial fibers with sucrose (PVA/Su PVP + phage) exhibited the highest viability (14.50%). Stability tests indicated storing the fibers at −20 °C resulted in minimal titre loss after 6 months of storage. However, notable titre loss was detected after the phage‐loaded fibers were stored at 4 °C and room temperature. Coaxial fibers did not provide better protection than monoaxial fibers during long‐term storage. Phage‐loaded coaxial fibers had a broad size distribution and larger diameters than monoaxial fibers. PVA/Su + phage monoaxial and PVA/Su PVP + phage coaxial fibers demonstrated immediate release profiles, with most phages released within 10 min. PVA/Su PVP/EC + phage core/shell fibers extended the release up to 2 hours. The antibacterial activity of the loaded fibers was studied using isothermal calorimetry and found to be retained after electrospinning. Thus, phage‐loaded electrospun fibers have the potential for the development of wound dressings for treating antibiotic‐resistant infections. Future work will focus on improving the antibacterial efficacy of electrospun fibers by exploring other lytic phages (alone and in combination) and phage‐antibiotic combinations.

## Conflict of Interest

The authors declare no conflict of interest.

## Author Contributions

Tian Ju performed conceptualization, data curation, investigation, methodology, and visualization, and wrote, reviewed, and edited the final manuscript. Jixuan Li performed data collection, and wrote, reviewed, and edited the final manuscript. Andrew Weston performed investigation, and wrote, reviewed, and edited the final manuscript. Giovanni Satta performed conceptualization, investigation, and methodology, and wrote, reviewed, and edited the final manuscript. Sara Bolognini performed methodology and investigation. Mariagrazia Di Luca performed investigation, and methodology, wrote, reviewed, and edited the final manuscript. Simon Gaisford performed methodology, and supervision, wrote, reviewed, and edited the final manuscript. Gareth R. Williams performed conceptualization, data curation, project administration, and supervision, and wrote, reviewed, and edited the final manuscript.

## Supporting information



Supporting Information

## Data Availability

The data that support the findings of this study are available from the corresponding author upon reasonable request.
