# Supplementary material for: Anti‐Pseudomonas Aeruginosa Bacteriophage Loaded Electrospun Fibers for Antibacterial Wound Dressings
Source: Macromol Rapid Commun. 2025 Jan 13;46(13):2400744. doi: 10.1002/marc.202400744 (PMC12227222; doi:10.1002/marc.202400744)
Supplement: Supplementary file 1 — Supporting Information [file MARC-46-2400744-s001.docx]

**Supplementary information for:**

Tian Ju^a^, Jixuan Li^a^, Andrew Weston^a^, Giovanni Satta^b^, Sara Bolognini,^c^ Mariagrazia Di Luca^c^, Simon Gaisford^a^, Gareth R. Williams^a^*

^a^ UCL School of Pharmacy, University College London, 29-39 Brunswick Square, London, WC1N 1AX, UK.

^b^ Centre for Clinical Microbiology, University College London, Royal Free Campus, Rowland Hill Street London, NW3 2PF, UK

^c^ Department of Biology, University of Pisa, Via San Zeno 39 56127 Pisa, Italy

Corresponding author: g.williams@ucl.ac.uk

ORCID: Tian Ju (0009-0006-7623-9132); Jixuan Li (0009-0008-1283-4228); Andrew Weston (0009-0006-6492-989X); Giovanni Satta (0000-0002-0120-7885); Sara Bolognini (0009-0007-4574-4512); Mariagrazia Di Luca (0000-0002-0688-034X); Simon Gaisford (0000-0003-1000-3208); Gareth R. Williams (0000-0002-3066-2860)

Keywords: Bacteriophage, coaxial electrospinning, *Pseudomonas. aeruginosa*, polyvinyl alcohol (PVA), polyvinylpyrrolidone (PVP), ethyl cellulose (EC), isothermal calorimetry

## **Calculation for viability of phage in electrospun fibres**

An example for PVA/Su PVP coaxial fibres

Phage lysate titer: 1.61×10^10^ PFU/mL

Polymer mass in 1 mL of solution: 599.2 mg

0.2 mL of phage lysate was added in 0.8 mL of polymer solution, therefore:

Phage per mg of fibres = 1.61×10^10^ PFU/mL × 0.2 mL / 599.2 mg = 5384957.72 PFU/mg

5.88 mg of fibres were immersed in 1 mL SM buffer.

Total phage in 5.88 mg of fibres: 5.88 mg × 5384957.72 PFU/mg = 31663551.40 PFU

Viable Phage released from fibres: 4800000 PFU/mL × 1 mL = 4800000 PFU

Viability = 4800000 PFU / 31663551.40 PFU × 100% = 15.16%

## **Thermal analysis**

### Thermogravimetric analysis (TGA)

Figure S1. TGA curves for raw materials and electrospun fibres.

### Differential scanning calorimetry (DSC)


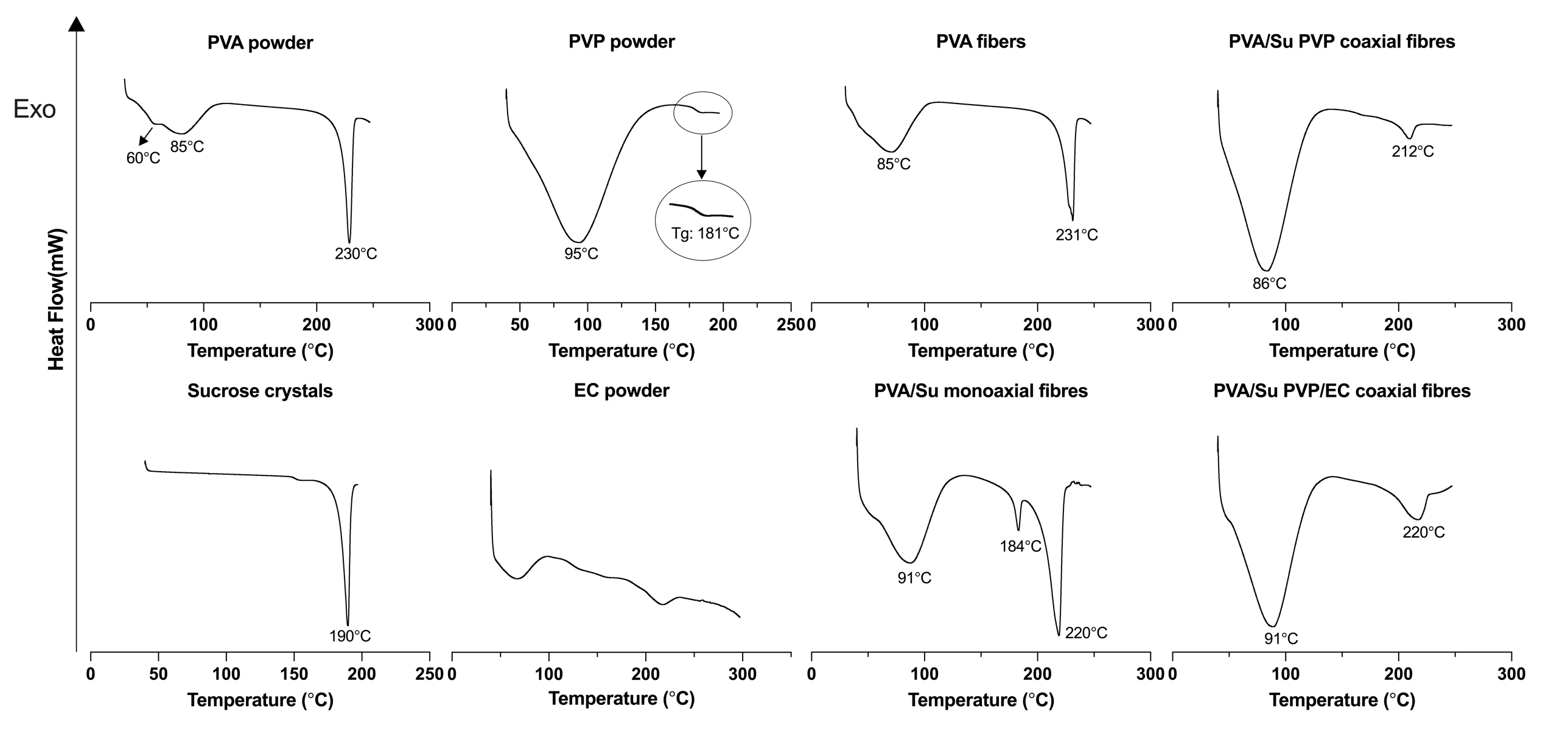


Figure S2. DSC thermograms of the raw materials and placebo fibres. PVA powder shows an enthalpy relaxation peak at 60 °C, a water evaporation endotherm at 85 °C and a melting endotherm at 230 °C [1]. The enthalpy relaxation peak corresponds to the release of heat after materials have been stored under the glass transition temperature. This, with the observation of a melting point, indicates that PVA is semicrystalline. PVP is amorphous. It has a broad water evaporation endotherm at 95 °C and a glass transition temperature (T_g_) at 181 °C [2]. Sucrose exhibits a sharp endotherm at 190 °C [3], revealing its crystalline nature. EC does not show any sharp endotherms or obvious shift of baseline. No apparent water evaporation endotherm can be found in DSC thermograms of sucrose and ethyl cellulose, which infers that they are dry. PVA fibres display a water evaporation endotherm at 85 °C and a sharp melting endotherm at 231 °C. PVA/Su monoaxial fibres show a broad water evaporation endoderm at 91°C, and two melting endotherms at 184 °C and 220 °C, which corresponds to the melting of sucrose and PVA respectively. However, the melting temperature drops 6 °C for sucrose and 10°C for PVA compared to the raw materials. This is expected: the melting temperature of blends is always lower than the pure materials [4, 5]. Thermograms of PVA/Su PVP coaxial fibres have a water evaporation endotherm at 86 °C and a melting endotherm from PVA at 212 °C. The reduced PVA melting temperature may be caused by interchain hydrogen bonding between PVA and PVP [6] destabilising the crystal lattice structure of PVA. Similar observations were reported in a previous study that fabricated PVA/PVP-iodine electrospun fibres [7]. PVA/Su PVP/EC coaxial fibres demonstrate a water evaporation endotherm at 91 °C and a melting endotherm of PVA at 220 °C. The melting temperature of PVA is the same as for the PVA/Su monoaxial fibres. No sucrose melting endotherms can be observed in the coaxial fibres.

## **X-ray diffraction (XRD)**

##

Figure S3. XRD patterns of the raw materials and placebo electrospun fibres. PVA powder is semicrystalline: its XRD pattern shows a broad halo with two sharp peaks at 21° and 23° [8]. PVP powder displays a broad halo [2], suggesting its amorphous nature. Sucrose is crystalline, which is illustrated by a number of well-defined Bragg reflection [8]. Ethyl cellulose is semicrystalline, with a broad halo and a sharp peak at 11° [9]. PVA monoaxial fibres have a broad halo with a characteristic peak at 20°, very different to the pattern of the PVA powder. This observation indicates very significantly reduced crystallinity after electrospinning, which arises from the rapid evaporation and solidification process [10]. PVA/Su monoaxial fibres exhibit a broad halo with a characteristic peak at 21°. No sharp Bragg reflections of sucrose can be found, implying that sucrose is amorphous. The DSC thermograms of both PVA monoaxial fibres and PVA/Su monoaxial fibres show the melting endotherm of PVA and sucrose, however: this apparent contradiction can likely be attributed to crystallisation occurring upon heating in the DSC experiment. PVA/Su PVP coaxial fibres exhibit a broad halo with two characteristic bands at 12° and 23° that can be ascribed to the PVP and PVA. Compared to PVA/Su PVP coaxial fibres, PVA/Su PVP/EC coaxial fibres only have a characteristic peak at 22°. These systems all appear to be amorphous.

## **Infrared spectroscopy (FTIR)**

Figure S4. FTIR spectra of the raw materials and placebo fibres. PVA presents peaks at 3240 cm^-1^ (-OH stretching), 2901 cm^-1^ (-CH stretching), and 1411 cm^-1^ (-OH bending) [8]. Sucrose has peaks at 3303 cm^-1^ (-OH stretching), 2913 cm^-1^ (-CH stretching) and 1048 cm^-1^  (C-O-C vibrations) [8, 11]. PVP exhibits peaks at 3386 cm^-1^ (-OH stretching from adsorbed water), 2921 cm^-1^ (-CH stretching) and 1646 cm^-1^ (C=O stretching) [2]. EC has peaks at 3443 cm^-1^ (-OH stretching), 2971-2865 cm^-1^ (-CH stretching), and 1050 cm^-1^ (C-O-C vibrations) [9]. The PVA and PVA/Su monoaxial fibres have similar FTIR spectra to PVA powder. However, while the PVA monoaxial fibres have a peak at 1085 cm^-1^, the PVA/Su fibres have a peak at 1045 cm^-1^. The observation of this shift might be attributed to the C-O-C vibrations of sucrose and confirms the existence of sucrose in PVA/Su monoaxial fibres. PVA/Su PVP coaxial fibres display peaks at 3266 cm^-1^ and 2915 cm^-1^, corresponding to -OH and -CH stretching of PVA and PVP. The peak at 1642 cm^-1^ is caused by C=O stretching of PVP. In comparison with PVA/Su PVP, PVA/Su PVP/EC coaxial fibres have an additional peak at 1051 cm^-1^ arising from the C-O-C vibrations of EC, confirming its presence in the fibres.

## **Broth dilution**

Figure S5. Antibacterial efficacy of the Neko phage at different concentrations (MOI 1/10/100) when tested using broth dilution. The percentage of bacteria reduction for all the concentrations is less than 40%.

# 2. References

[1] M. Razzak, S. Dewi, H. Lely and E. Taty, Radiat. Phys. Chem. **1999**, 55, 153-165.

[2] Y. Geng, F. Zhou and G. R. Williams, J. Drug Deliv. Sci. Technol. **2021**, 61, 102138.

[3] M. Hurtta, I. Pitkänen and J. Knuutinen, Carbohydr. Res. **2004**, 339, 2267-2273.

[4] S. Aid, A. Eddhahak, S. Khelladi, Z. Ortega, S. Chaabani and A. Tcharkhtchi, Polym. Test. **2019**, 73, 222-231.

[5] G. Groeninckx, C. Harrats, M. Vanneste and V. Everaert, "Crystallization, Micro- and Nano-structure, and Melting Behavior of Polymer Blends", in Polymer Blends Handbook, L. A. Utracki and C. A. Wilkie, Eds., Springer Netherlands, Dordrecht, 2014, p. 291-446.

[6] N. Roy and N. Saha, Hydrogels: Synthesis, Characterization and Applications **2012**, 227-252.

[7] F. Gökmeşe, I. Uslu and A. Aytimur, Polym.-Plast. Technol. Eng. **2013**, 52, 1259-1265.

[8] K. Katopodis, A. Kapourani, E. Vardaka, A. Karagianni, C. Chorianopoulou, K. N. Kontogiannopoulos, D. N. Bikiaris, K. Kachrimanis and P. Barmpalexis, Int. J. Pharm. **2020**, 578, 119121.

[9] Y. Geng and G. R. Williams, Int. J. Pharm. **2023**, 648, 123557.

[10] V. Thomas, M. V. Jose, S. Chowdhury, J. F. Sullivan, D. R. Dean and Y. K. Vohra, J. Biomater. Sci. Polym. Ed. **2006**, 17, 969-984.

[11] M. Beekes, P. Lasch and D. Naumann, Vet. Microbiol. **2007**, 123, 305-319.
